# ASSESSING RELIABILITY AND AGREEMENT IN TOPOGRAPHIC MEASUREMENT OF RETICULAR PSEUDODRUSEN AREA: A Multimodal Imaging Approach

**DOI:** 10.1097/IAE.0000000000004514

**Published:** 2025-05-07

**Authors:** Mariano Cozzi, Andrea Trinco, Francesco Romano, Sandrine A. Zweifel, Giovanni Staurenghi, Alessandro Invernizzi

**Affiliations:** * Department of Biomedical and Clinical Sciences, Eye Clinic, University of Milan, Milan, Italy;; † Department of Ophthalmology, University Hospital Zurich, University of Zurich, Zurich, Switzerland;; ‡ Harvard Retinal Imaging Lab, Retina Service, Department of Ophthalmology, Massachusetts Eye and Ear, Harvard Medical School, Boston, Massachusetts;; § Faculty of Health and Medicine, Save Sight Institute, University of Sydney, Sydney, NSW, Australia.

**Keywords:** fundus autofluorescence (FAF), intermediate age-related macular degeneration (iAMD), near-infrared reflectance (NIR), optical coherence tomography (OCT), reticular pseudodrusen (RPD), subretinal drusenoid deposits (SDD)

## Abstract

**Purpose:**

This study aims to assess the reliability and agreement of fundus autofluorescence, near-infrared reflectance (NIR), and a combination of NIR and dense structural optical coherence tomography (OCT) scans (OCT + NIR) in delineating the areas of reticular pseudodrusen (RPD) in eyes affected by age-related macular degeneration.

**Methods:**

This was a single-center, cross-sectional study. Patients with nonadvanced age-related macular degeneration exhibiting signs of RPD on multimodal imaging were enrolled. Two independent masked graders manually delineated the margins of the area occupied by RPD using the three distinct imaging techniques.

**Results:**

The study included 60 eyes from 51 patients, with a mean age of 81.5 (±7.1) years. The intraclass correlation coefficient between the two graders across all imaging modalities was 0.96 for fundus autofluorescence, 0.92 for NIR, and 0.98 for OCT + NIR. The narrowest limits of agreement were observed with OCT + NIR (−4.38 + 5.17 mm^2^). Foveal involvement and age were significantly correlated with larger RPD area (*P* = 0.036 and *P* = 0.019, respectively). Pairwise comparisons of square root-transformed RPD areas indicated that fundus autofluorescence detected a significantly larger RPD area compared with other methods (*P* < 0.001).

**Conclusion:**

These findings validate OCT + NIR as a reliable approach for measuring RPD areas, potentially serving as a critical biomarker for age-related macular degeneration in future clinical trials.

## Abstract 2

Supplemental Digital Content is Available in the Text.

Combined near-infrared reflectance and optical coherence tomography seems the most reliable imaging approach to assess topographic distribution and area of reticular pseudodrusen, a potential quantitative marker for age-related macular degeneration progression.

Reticular pseudodrusen (RPD), also known as subretinal drusenoid deposits, are characterized by the accumulation of heterogenous material located between the photoreceptor outer segments and the retinal pigment epithelium (RPE) on spectral domain optical coherence tomography.^1,2^ These deposits present a distinct pattern that mirrors the distribution of rod photoreceptors, typically exhibiting a higher density in the parafoveal region while sparing the fovea.^3–5^ This topographic distribution explains the direct link between RPD and impaired rod-associated visual function, highlighting their clinical significance.^6^

According to the most commonly used clinical classification of age-related macular degeneration (AMD), based on color fundus photography, RPD is notably absent from the disease spectrum.^7^ However, over the past decade, multimodal imaging–based studies have reported their prevalence ranging from 26% to 79% in eyes with early/intermediate AMD and established an association between RPD/subretinal drusenoid deposit's and AMD progression.^8–10^ This association is particularly pronounced when the fellow eye is afflicted by neovascular AMD.^11,12^ Recently, a report from the age-related eye disease study 2 identified RPD as the third independent risk factor for the progression to late AMD.^13^

Color fundus photography has been recognized as an unreliable modality for revealing RPD.^9,14^ In contrast, confocal near-infrared reflectance (NIR) and fundus autofluorescence (FAF) have proven to be more effective imaging modalities to identify these lesions.^1,15–18^ Beyond the detection and topographic distribution, effective identification of RPD/subretinal drusenoid deposit's frequently requires the use of OCT images, with the simultaneous consultation of *en face* images and structural scans being proposed as essential diagnostic criterion for comprehensive RPD characterization.^2,19^

Historically, studies have predominantly assessed RPD qualitatively, reporting their presence or absence in each eye, with a limited number of studies delving into their topographic distribution. Reports focusing on the quantification of the area affected by RPD are significantly limited. Recently, Wu et al^20^ used the simultaneous detection of RPD using NIR and OCT to calculate the RPD area in a 6 × 6-mm volume scan. This approach was further applied by the same group in a subsequent study.^21^ More recently, Duic et al^22^ adopted a similar method, using a wider OCT scan pattern. Although the combination of NIR and OCT seems the most commonly used method for topographically assessing the RPD area, it is important to note that this approach has not yet undergone validation or comparison with other imaging modalities.

This study aims to achieve two primary objectives. First, to evaluate the reliability and agreement of RPD area measurements using three different imaging modalities: NIR, FAF, and the combined OCT + NIR. These measurements will be conducted by two independent graders in a population with nonadvanced AMD exhibiting RPD. Second, we aimed to determinate differences in RPD area between the three imaging modalities.

## Methods

This single-center, cross-sectional study was conducted at the Eye Clinic of Luigi Sacco Hospital (Department of Biomedical and Clinical Sciences, University of Milan) and received approval from the Milano Area 1 institutional review board. The research adhered to the principles outlined in the Declaration of Helsinki, and written informed consent was acquired from all eligible participants upon enrollment.

### Population

Consecutive patients with diagnosis of early or intermediate AMD in either eye with the presence of RPD based on the multimodal imaging evaluation were considered eligible for the study. Detailed inclusion criteria were as follows: 1) age >55 years; 2) presence of RPD at the posterior pole confirmed by multimodal imaging; 3) best-corrected visual acuity greater than 20/32; 4) overall Heidelberg OCT scan quality greater than 25 db; 5) refractive error (spherical equivalent) between −6 and +3 diopters; and 6) clear ocular media. Eyes with advanced AMD, either neovascular or geographic atrophy, or any other macular alterations (e.g., glaucoma and diabetic retinopathy) were excluded from the analysis.

### Reticular Pseudodrusen Imaging Definition

**Fig. 1.**
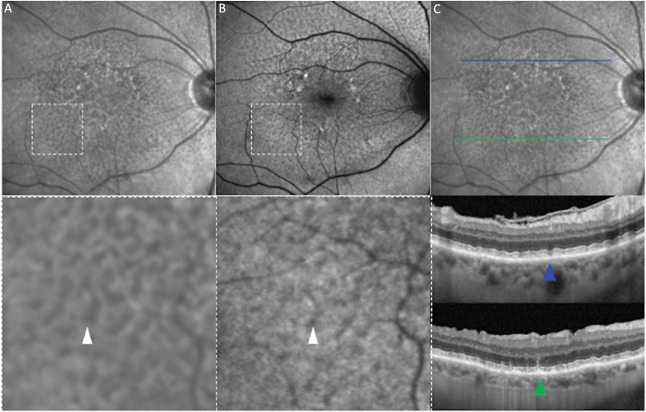
RPD imaging definition. Multimodal imaging was used to identify the presence of RPD. **A.** With a magnified inset, shows a NIR image depicting a network of hyporeflective lesions against a mildly hyperreflective background. In the same area, imaged with FAF, a reticular pattern of lesions is visible, characterized by decreased autofluorescence, surrounded by areas of increased autofluorescence (**B**). Finally, in panel (**C**), OCT B-scans differentiate two RPD subtypes: Ribbon RPD, appearing as medium-reflective mounds elevating the ellipsoid zone and external limiting membrane without disrupting their integrity (blue arrowhead), and dot RPD, characterized by a conical shape protruding through the ellipsoid zone (green arrowhead).

Reticular pseudodrusen were defined according to the classification system proposed by Zweifel et al, and Spaide et al as the presence of at least five definite subretinal drusenoid lesions observed on a minimum of one OCT B-scan, further confirmed by at least one *en face* imaging modality, either FAF or NIR.^2,14,19^ On OCT, RPD were defined as hyperreflective abnormalities or elevations above the RPE/Bruch's membrane complex, with medium-reflective mounds or cones at the level of the ellipsoid zone or between the ellipsoid zone and the RPE surface.^1,17,18^ On NIR, RPD were defined as a network of hyporeflective lesions against a mildly hyperreflective background that may have a hyperreflective or isoreflective core.^17,23,24^ On FAF, RPD were defined as a reticular pattern of lesions characterized by decreased autofluorescence signal, surrounded by areas of increased autofluorescence (Figure 1).^1,25^

### Imaging Examination

All enrolled subjects underwent a comprehensive ophthalmic examination, including best-corrected visual acuity, biomicroscopy, intraocular pressure assessment, and funduscopic examination.

Before the imaging session, participant's eyes were dilated with a combination of 0.5% tropicamide and 2.5% phenylephrine. The multimodal imaging protocol consisted of OCT scans, followed by NIR and FAF, with the latter always performed last to minimize bleaching and improve patient compliance. Imaging was conducted using a confocal scanning laser ophthalmoscope combined with OCT (Heidelberg Spectralis OCT2, Heidelberg Engineering GmbH, Heidelberg, Germany).

A dedicated 30° × 25° OCT volume, centered on the fovea, was acquired. The protocol comprised 121 horizontal B-scans with an interscan distance of 65 *µ*m. Each B-scan consisted of 1536 A-scans and underwent ninefold averaging (automatic real time = 9). In addition, two single-line 30° B-scans, one horizontal and one vertical, each comprising 100 averaged scans using enhanced depth imaging modality, were acquired to visualize the sclerochoroidal junction beneath the fovea.

Near-infrared reflectance and FAF images were captured using a 30° lens with a resolution of 1,024 × 1,024 pixel. Each *en face* image comprised a minimum of 30 averaged frames and was centered onto the fovea.

### Images Analysis

The overall clinical imaging set was initially graded by a single reader (F.R.), who reviewed the inclusion and exclusion criteria, confirmed the presence of RPD according to the study protocol, and evaluated the overall images quality. This grader also measured the subfoveal choroidal thickness on enhanced depth imaging single-line scans, evaluated foveal involvement—defined as the presence of RPD within the central 1-mm Early Treatment Diabetic Retinopathy Study circle—and recorded the presence of large drusen (≥125 *μ*m).

Subsequently, NIR and FAF images were imported into the open-source image processing software FIJI (Version 2.14, National Institutes of Health, Bethesda, MD) to standardize the area of interest before RPD measurement, accounting for differences in image dimensions (see **Figure 1, Supplemental Digital Content 1**, http://links.lww.com/IAE/C572). To achieve accurate spatial alignment between imaging modalities, we used the Data Science for Health plugin to coregister NIR and FAF images with the NIR image coacquired with the 30° × 25° OCT volume. Coregistration was performed by selecting a minimum of three anatomical landmarks, specifically large retinal vessel bifurcations or crossings, ensuring precise correspondence between modalities. To prevent geometric distortions, we applied a rigid transformation, allowing only translation, rotation, and uniform scaling. After successful coregistration, the NIR and FAF images were cropped to match the 30° × 25° region of interest corresponding to the OCT volume, superimposed onto the coacquired NIR image. Finally, RPD area was delineated on both NIR and FAF images using the freehand selection tool, following calibration of the spatial scale to 9 mm per 1,024 pixels for accurate quantification.

Each image was independently graded by two blinded graders (M.C., A.T.) to assess the area affected by RPD using the IR-only, FAF-only, and OCT + NIR modalities. To be included in the area calculation, RPD had to form a cluster of at least five distinct lesions in the *en face* modality. Near-infrared reflectance and FAF grading was conducted according to the above RPD definition. Each grader first identified the RPD clusters and subsequently delineated the boundaries to ensure the entire RPD-affected area was included.

**Fig. 2.**
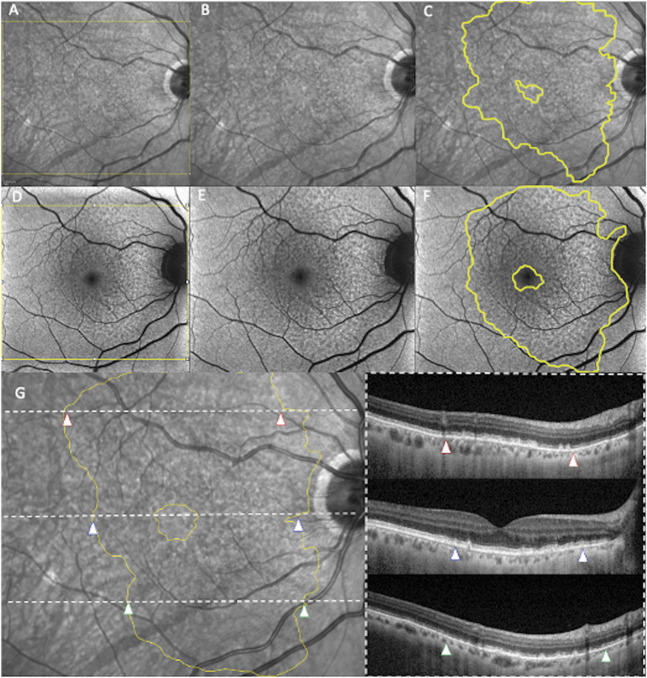
Images analysis. NIR and FAF images were acquired and imported into the open-source software FIJI (version 2.14, National Institutes of Health, Bethesda, MD) for analysis (**A** and **D**). The “crop” function was first applied to refine the area of interest to match the 30° × 25° OCT volume (**B** and **E**). Two independent graders then manually delineated the boundaries of the RPD area, excluding the fovea if it was not involved (**C** and **F**). The combination of OCT and NIR (**G**) served as the reference standard for assessing RPD. Using the in-built tools of the Heidelberg OCT2 Spectralis, the graders manually localized the RPD on the structural OCT scans and subsequently delineated the corresponding RPD area on the NIR images, indicated by red-, blue-, and green-framed arrowheads.

The simultaneous assessment by OCT + NIR served as the reference standard. In detail, each B-scan within a macular volume was individually assessed to determine the presence of RPD. Once elements considered part of the lesion area were identified, the grader manually outlined the area corresponding to the cluster of hyporeflective lesions on NIR. This process was facilitated by the in-built OCT reviewing software, which enabled topographical colocalization of OCT lesions on the corresponding NIR image (Figure 2). The RPD area was determined using the three distinct imaging modalities regardless the presence of drusen other than RPD. When drusen coexisted with RPD, the regions occupied by these drusen could have been included into the measured affected area in case they were placed between RPD.

For comparison between grading methods, the mean values of the two graders were used.

### Outcome Measures

The primary outcome was to assess the agreement between two independent graders in evaluating the extent of RPD. To quantify this agreement for each imaging modality, the intraclass correlation coefficient (ICC) was computed. In addition, Bland–Altman plots with limits of agreement (LoA) were used to further explore graders agreement for each imaging modality. The ICC was also applied to evaluate the agreement across different imaging modalities.

The secondary outcome was to determinate differences in RPD area between imaging modalities. The Kruskal–Wallis test was initially used to compare the RPD area across FAF, NIR, and OCT + NIR imaging modalities. Subsequently, to investigate the influence factors such as grader, RPD foveal involvement, subfoveal choroidal thickness, age, presence of drusen, and the imaging modality on the RPD area, a linear mixed-effects model was used.

### Statistical Analysis

The Shapiro–Wilk test was used to assess the normal distribution of the quantitative variables under study. Results are presented as mean ± SD for normally distributed continuous variables, or as median and interquartile range for non-normally distributed continuous variables. Categorical variables are summarized as counts and percentages, as appropriate.

In the linear mixed-effects model, the interactions between imaging modality and drusen and between imaging modality and foveal involvement were also tested. To improve fit the model and enhance the normality of residuals, a square root transformation of the RPD area was applied. The response variable was modeled as a function of the fixed effects of interest, which included the different factors under study and the random effects of patient ID and eye ID. After fitting the model, pairwise comparisons of the estimated marginal means for the three imaging modalities were performed. A Bonferroni adjustment was applied to control for the family-wise error rate across multiple comparisons. These comparisons aimed to identify significant differences in the square root-transformed RPD area between the three imaging modalities.

A *P*-value <0.05 was considered statistically significant. All statistical analyses were conducted; using R software version 4.1.2 (R Project—The R Foundation for Statistical Computing, Vienna, Austria).

## Results

A total of 60 eyes from 51 patients (36 female, 70.6%) with nonadvanced AMD and clinical evidence of RPD were enrolled in the study. The mean age of the group was 81.5 ± 7.1 years (range: 67–98 years). The median (and interquartile range) areas affected by RPD, as calculated by FAF, NIR, and OCT + NIR, were 24.17 (24), 21.9 (18.5), and 20.9 (22.5) mm^2^, respectively. Table 1 presents the demographic data and clinical characteristics of the eyes included in the analysis.

**Table 1.** Demographic and Clinical Characteristics

Number of eyes	60
Number of subjects	51
Age, years, mean (SD)	81.5 (7.1)
Female, n (%)	36 (70.6%)
Male, n (%)	15 (29.4%)
Laterality OD, n (%)	26 (43.3%)
Reticular pseudodrusen area	
FAF, mm^2^, median (IQR)	24.17 (24)
NIR, mm^2^, median (IQR)	21.9 (18.5)
OCT-NIR, mm^2^, median (IQR)	20.9 (22.5)
Imaging factors	
SCT, *μ*m, mean (SD)	167.9 (73.2)
Eyes with RPD only, n (%)	10 (16.7%)
Eyes with foveal-involvement RPD, n (%)	10 (16.7%)

IQR, interquartile range; OD, right eye; SCT, subfoveal choroidal thickness.

### Intergrader Agreement

**Fig. 3.**
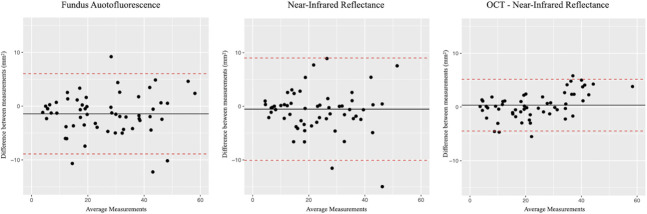
Intergraders agreement. Bland–Altman plots illustrating the intergrader agreement in the measurement of the RPD area using three different imaging modalities. The black lines represent the mean differences between the devices, while the dashed lines indicate the upper and lower LoA. The zero line denotes the absence of differences between the graders.

The agreement between the two graders was evaluated using the two-way ICC for all the imaging modalities under study. In particular, the ICC for FAF was 0.96 (95% confidence interval [CI] 0.93–0.98), for NIR, it was 0.92 (95% CI 0.88–0.95), and for OCT + NIR, the ICC was 0.98 (95% CI 0.97–0.99). A statistically significant difference in ICC values was observed only between NIR and OCT + NIR (adjusted *P* < 0.001). For each modality, the Bland–Altman plots were used to illustrate the agreement between the two graders along with the LoA calculated as the mean difference ± 1.96 times the SD of the differences (Figure 3). From the Bland–Altman plot, the OCT + NIR modality appears the one with the LoA closer to the zero (−4.38 + 5.17 mm^2^). Conversely, LoA for FAF and NIR were −8.82 + 5.97 mm^2^ and −10.00 + 8.89 mm^2^, respectively.

### Intermodality Agreement

The agreement across different imaging modalities was evaluated using the two-way ICC based on the mean RPD area between the two graders. Specifically, the ICC between NIR and FAF was 0.87 (95% CI: 0.77–0.93). The agreement between NIR and OCT + NIR was 0.93 (95% CI: 0.88–0.96), whereas the ICC between FAF and OCT + NIR was 0.92 (95% CI: 0.80–0.96). No statistically significant differences in ICC values were detected between modalities (all adjusted *P* > 0.05).

### Imaging Modalities Comparison

The Kruskal–Wallis test did not reveal any significant differences in RPD area across FAF, NIR, and OCT + NIR imaging modalities (*P* = 0.32). However, further analysis using pairwise comparisons on the square root-transformed RPD area showed that FAF detected a significantly larger RPD area compared with both NIR and OCT + NIR (*P* < 0.001). Conversely, the contrast between OCT + NIR and NIR was not significant (*P* =1.00), suggesting almost identical RPD area measurements between these two modalities (Table 2).

**Table 2.** Multiple Regression Analysis With Pairwise Comparison

Contrast	Mean Difference (Estimate)	SE	Lower 95%	Upper 95%	Corrected *P*
FAF − NIR	0.2211	0.0512	0.08	0.36	**<0.001**
OCT + NIR − FAF	−0.2613	0.0512	−0.40	−0.13	**<0.001**
OCT + NIR − NIR	−0.0402	0.0512	−0.18	0.01	1.000

Results are averaged over the levels of grader, foveal involvement, and drusen. Estimates are presented on the square root scale. *P* value adjustment: Bonferroni method for three tests. Significant *P* values (*P* < 0.05) appear in bold.

### Estimated Reticular Pseudodrusen Area

In the linear regression analysis evaluating the effects of various variables on the square root-transformed RPD area, we found that foveal involvement (β = 0.97, *P* = 0.04), age (β = 0.06, *P* = 0.02), and the interaction between presence of drusen and FAF imaging modality (β = 0.40, *P* = 0.003) were significantly associated with a greater RPD area (Table 3). Notably, the use of the FAF imaging modality in cases of RPD associated with drusen resulted in an increased RPD area detection, with a mean difference of 0.40 units.

**Table 3.** Results of the Linear Mixed-Effects Model Applied to the Square Root of the RPD Area

Predictors	sqrt (RPD Area)		
	Estimates	**Confidence Interval**	*P*
Intercept	−0.55	−5.04 to 3.94	0.810
Grader (M.C.)	−0.08	−0.16 to 0.00	0.066
Foveal involvement (yes)	0.97	0.06 to 1.88	**0.036**
Subfoveal choroidal thickness	−0.00	−0.01 to 0.00	0.539
Age	0.06	0.01 to 0.11	**0.019**
Drusen (yes)	0.30	−0.23 to 0.83	0.269
Imaging (FAF)	−0.06	−0.31 to 0.19	0.636
Imaging (NIR)	−0.04	−0.29 to 0.21	0.774
Drusen (yes):imaging (FAF)	0.40	0.14 to 0.67	**0.003**
Drusen (yes):imaging (NIR)	0.09	−0.18 to 0.24	0.821
Imaging (FAF):foveal involvement (yes)	−0.03	−0.30 to 0.24	0.821
Imaging (NIR):foveal involvement (yes)	0.04	−0.23 to 0.30	0.788
Random effects			
σ^2^	0.15		
τ00 Eye_ID:Pt_ID	0.34		
τ00 Pt_ID	1.07		

The model includes 60 eyes from 51 patients. The interaction between drusen and imaging (drusen:imaging) and between imaging and foveal involvement (imaging:foveal involvement) has also been tested. Significant *P* values (*P* < 0.05) appear in bold.

## Discussion

In this study, we demonstrated that combining structural OCT dense volume scan with NIR images offers the most reliable technique for accurately measuring the areas of the posterior pole affected by RPD in eyes with nonadvanced AMD. These findings highlight the advantage of this combined approach for providing a more comprehensive assessment of their spatial distribution compared with FAF and NIR alone.

Historically, detecting RPD using standardized color fundus photography has been challenging, with more than half of cases being missed when compared with OCT detection.^9^ As a result, more advanced *en face* imaging modalities have emerged to overcome the low sensitivity of color fundus photography. However, the RPD detection remains largely limited to identifying the presence or absence of lesions, with little attention given to assessing the extent of RPD affected areas.

The agreement between the two graders in our study was excellent across all three imaging modalities (all ICC > 0.90), indicating a high level of concordance in the measurements. However, Bland–Altman plots revealed narrower LoA when combining structural OCT with NIR images, thus supporting this approach as the most reliable for defining the RPD area because of the lower intergrader variability. Previous research has explored interreader agreement for RPD assessment using combined OCT + NIR, involving 12 readers from 6 reading centers.^26^ The study by Wu et al showed a generally substantial, although not perfect, interreader agreement in identifying RPD presence on OCT, whether from entire volume scans or selected B-scans. Importantly, this previous research did not assess the area affected by RPD. Similarly, other studies have examined agreement between graders in identifying RPD using structural OCT, which is relevant for the combined OCT + NIR approach, but these findings are not directly comparable to ours.^16,27^

An early attempt to combine structural OCT and NIR for the detection of RPD was initially described in 2010 by Zweifel et al.^1^ Schmitz-Valckenberg et al^24^ aimed to study the alterations of individual RPD lesions over time using OCT and NIR. They used a smaller, dense scan pattern to measure RPD height but did not provide an evaluation of the topographic distribution. More recently, Kumar et al^21^ adopted a simultaneous combination of OCT and NIR to investigate the impact of RPD extent on retinal sensitivity assessed on mesopic microperimetry. Their approach involved a 20° × 20° OCT volume scan, centered on the macular area. The same research group applied a similar methodology to examine the potential association between RPD spatial extent and AMD progression but found no significant correlation.^20^

In our study, we opted for a larger volume scan (30° × 25°) with a dense scan pattern consisting of 121 B-scans. The larger scan size, combined with a greater number of B-scans, allows for more precise detection of RPD on OCT and covers a broader area that may be affected by these lesions. The same combined OCT and NIR approach investigated in our study was used by Duic et al^22^ in a longitudinal study evaluating the spatial distribution of RPD over time. Notably, none of the mentioned studies reported intragrader agreement in the detection of RPD areas.

Fundus autofluorescence has been successfully used in previous studies to evaluate the presence of RPD in patients with AMD.^15,16,28^ Steinberg et al^29^ used FAF to delineate the boundaries of the RPD area in 36% of eyes with visible RPD, demonstrating good interobserver agreement in cases with clearly defined RPD extent. This methodology was later used to determine the longitudinal structure–function correlation with multifocal electroretinogram in eyes with RPD, with the study reporting a mean RPD area growth rate of 3.3 mm^2^ per year.^30^ In our study, we found excellent intraobserver agreement between graders in detecting the RPD area. However, pairwise comparisons revealed that the FAF-measured area was significantly larger than the area measured with either NIR or OCT + NIR. A possible explanation for this overestimation could be that altered RPE fluorescence in AMD could complicate the detection of RPD area boundaries.

The involvement of RPD within the central millimeter of the Early Treatment Diabetic Retinopathy Study foveal region was a significant determinant of lesion extension, regardless of the imaging method used. While macular pigment affects short-wavelength FAF, NIR imaging can also be influenced by reflections in the foveal area, potentially affecting the detection of RPD. Moreover, the spatial distribution of RPD is more likely to affect the perifoveal region, which have a higher density of rods. Advanced stages of RPD extension might affect also the central macular area.

**Fig. 4.**
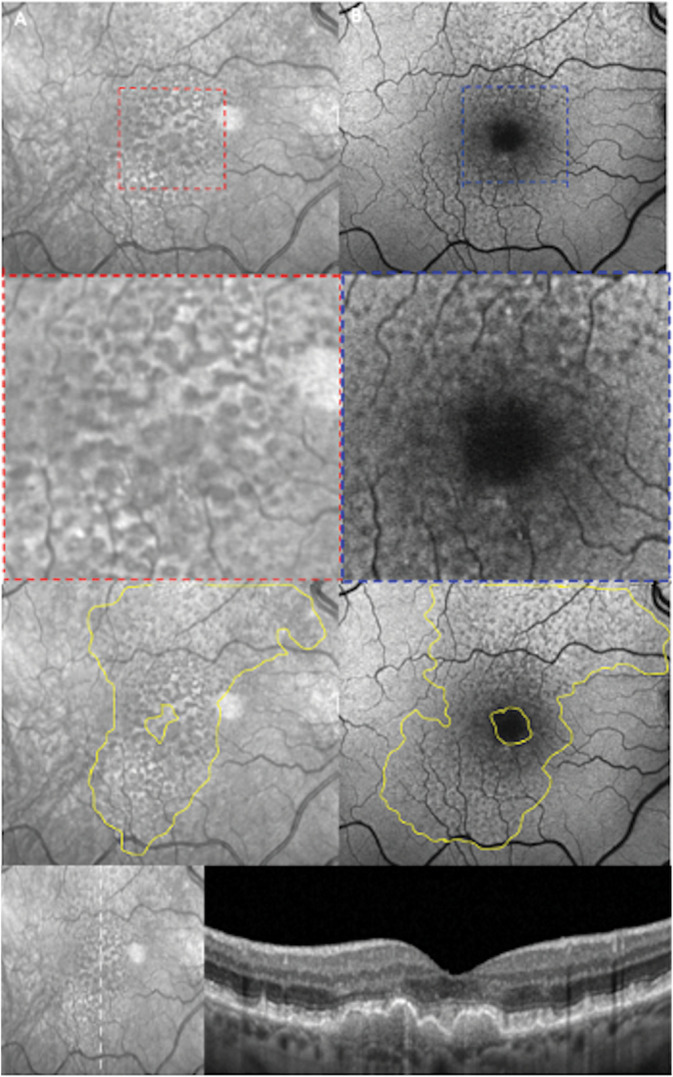
Comparison of en face modalities in the detection of RPD area in patient with intermediate AMD. Notable differences between NIR and FAF in detecting RPD area are evident in both foveal and macular region. In the absence of reflection artifacts, NIR provides clear visualization of the macula (**A**), facilitating precise identification of involvement by drusen or RPD (red dashed box). Conversely, FAF images (**B**) are hampered by the presence of macular pigment (blue dashed box) and drusen-related altered autofluorescence, which compromises the accurate analysis of the affected area. The bottom panel illustrates a vertical B-scan through the fovea, highlighting the concurrent involvement of the fovea by both RPD and drusen, a condition associated with larger RPD areas when measured with FAF.

In addition, we identified the interaction between FAF measurements and the presence of drusen as a factor that significantly influenced the size of the RPD area. Drusen are known to alter the autofluorescence signal, complicating the identification and precise quantification of the typical RPD autofluorescence pattern (Figure 4).

Another key factor that showed a significant correlation with the RPD area was age, consistent with studies reporting an increased prevalence of RPD in older individuals. Specifically, the prevalence of RPD is estimated to be around 50% in patients over the age of 85.^31^

Drusen, a hallmark feature of AMD, are typically classified based on their clinical and imaging characteristics and can be quantified using drusen volume. This measurement is easily obtained with most built-in OCT software, which calculates the distance between Bruch membrane and the RPE layer for each B-scan. Drusen volume has been shown to be an accurate and reproducible biomarker associated with an increased risk of developing advanced AMD.^32–34^ In contrast, determining the volume of RPD presents significant challenges because of their much thinner structure compared with drusen, making automatic volumetric OCT-based segmentation of the upper boundary difficult. To address this, OCT-based deep learning frameworks have been developed, achieving substantial interrater agreement in the automatic detection and quantification of RPD.^35^ In addition, structural *en face* OCT has been used to analyze the topographic distribution of RPD, revealing an interesting trizonal distribution of drusen and RPD. Macular drusen were found primarily located in the central Early Treatment Diabetic Retinopathy Study circle, while dot and ribbon RPD predominantly appeared in the inner and outer Early Treatment Diabetic Retinopathy Study rings, respectively.^36^

Our study has important limitations that need to be addressed. First, a larger sample size with longitudinal data will be necessary in the future to confirm our findings. Second, we intentionally avoided characterizing the RPD spatial profile, as previously reported in other studies. Although this information is certainly of interest, the study was designed with a different aim. Third, we did not collect data on RPD counting and density, which is challenging to obtain through manual evaluation alone. In the future, artificial intelligence–based tools may facilitate the measurement of RPD density within specific areas.

In conclusion, we demonstrated the reproducibility and agreement of different methodologies for topographically delineating the macular extension of RPD. Our data support the combined use of structural dense volume OCT and NIR for the manual definition of the RPD area. In this context, the RPD area may serve as an important anatomical biomarker for longitudinal evaluation in clinical trials focusing on nonadvanced AMD. Further investigations with long-term follow-up and the incorporation of artificial intelligence algorithms are warranted to explore this biomarker in greater depth.
